# The effectiveness of video-based exercise training program for people with intellectual disability: a multicenter study

**DOI:** 10.3389/fspor.2024.1388194

**Published:** 2024-05-09

**Authors:** Rachel Kwan, Grace Szeto, Ester Ho, Annie Wu, Lavinia Wong, Gilbert Ho, Rufina Lau, Eyckle Wong, Anthony Kwok, Dorothy Cheung

**Affiliations:** School of Medical and Health Sciences, Tung Wah College, Kowloon, Hong Kong SAR, China

**Keywords:** exercise, intellectual disability, multicenter study, physical performance, balance

## Abstract

**Objective:**

To examine the effectiveness of a specially designed video-based exercise program in promoting physical and balance performance in people with intellectual disability.

**Methods:**

This study was a multicenter controlled trial. Participants with intellectual disability were divided into exercise group and control group by cluster sampling. The participants in the exercise group received 1 h exercise training sessions twice a week for 8 weeks, and the controls continued their usual care without exercise training. The exercises were specially designed to match the physical ability level of the participants classified as high and low, and a third group called “special” was designed for those wheelchair-bound persons with limited mobility. Elements of light-tempo music and animation were introduced in the videos to motivate the participants. Recording the exercises in video format makes it easier for the class instructors and participants to perform the exercises together, and ensure consistency across different exercise groups conducted in different centers. Each participant underwent the pre- and post-intervention assessment including 30-s chair stand repetitions, five-time chair stand duration, 4-m comfortable walk time, standing static balance level, 6-min walk test, and short physical performance battery score. These variables were compared within each group at pre- and post-intervention stages, and they were also compared between the two groups.

**Results:**

A total of 180 participants were enrolled in 16 subcenters, including 160 participants in the exercise group and 20 participants in the control group. After 8 weeks of exercise training, there were significant improvements in their physical performance including 30-s chair stand repetitions and five-time chair stand duration, 4-m comfortable walk time and also 6-min walk test, within the exercise group (all *P *< 0.05). Approximately 39% of the participants in the exercise group also showed significant improvement in standing static balance level. No significant differences were found when compared with the control group participants who did not have any regular exercise participation.

**Conclusion:**

A specially designed video-based exercise program has demonstrated some positive effects on physical and balance performance after 8 weeks of training among adults with intellectual disability.

## 1 Introduction

People with intellectual disability (ID) exhibit many physical impairments that affect their daily functions. It has been reported that they have consistently lower levels of physical fitness and balance performance compared with the age-matched healthy population (1, 2). People with ID often adopt a sedentary lifestyle and lack motivation to exercise, resulting in obesity, poor fitness, and secondary health problems (3). The prevalence of multimorbidity in adults with intellectual disabilities is 98.7%, including visual impairment, obesity, epilepsy, constipation, and gait disorders (4). In particular for those aged 40 years and above, aging in people with ID is associated with low physical fitness and high prevalence of chronic diseases (5).

Falls are highly prevalent in people with ID causing severe injuries such as bone fractures (6). Physical deficit significantly contributes to health problems of people with ID by reducing their ability or motivation to participate in physical activity (7). Their inactive lifestyles may further lower their physical capacities and function, which also affect balance resulting in a vicious cycle. A meta-analysis has shown that exercise can significantly improve both static and dynamic balance in people with ID (8).

While there have been some studies in the research literature on the effects of exercise training among people with ID, the evidence is limited and varied. It is indeed a challenge to motivate people with ID to exercise on a regular basis, hence there is a need to make exercise entertaining and interesting to encourage their participation. It is also important to incorporate different components such as cardiovascular, strength training, flexibility, coordination, and balance in the same training program. Multi-component exercise training can improve functional independence, muscle strength, and balance of people with ID and promote better quality of life (9).

When designing exercises for people with ID, it is important to keep the movements and instructions simple and straightforward. Using music and animation to produce exercise videos can be effective in motivating the clients to engage in the exercises actively. Rhythmic movements with music were shown to improve the attention and concentration of students with intellectual disability (10). Using a light-tempo music helps to make the exercises more fun and motivate the participants to follow. These elements provide sensory cues that will stimulate the sensorimotor system of the participants and may help to facilitate their movements and coordination. This may have a positive benefit on their ability to perform physical movements in their daily lives. These sensory cues also serve to improve the attention focus of the participants. By repeating the same video clips, the participants also become more familiar with the exercises. Developing an exercise program with input from different healthcare professionals such as physiotherapists, occupational therapists, and healthcare workers can generate creative ideas that can address the specific needs of the people with ID.

The objective of the present study is to develop a special video-based exercise program with added elements of music and animation that may benefit those individuals with ID at high and low fitness levels. The aim is to enhance their active lifestyle and improve their overall fitness and health conditions. It is hoped that such a training program can be adopted in different community-based organizations that look after people with intellectual disabilities.

## 2 Materials and methods

### 2.1 Study design

This was a multicenter controlled trial to evaluate the effectiveness of an exercise program for people with intellectual disabilities.

This study was approved by the Research Ethics Committee of the Tung Wah College, Hong Kong, China. Each participant and their caregiver were informed of the purpose and procedures of the study and signed the consent before the study began.

### 2.2 Participants

The organizations that provided care for people with ID were invited to participate in this project. The person-in-charge of the center was responsible for identifying individuals who were suitable to join the training program. People over 15 years of age diagnosed with intellectual disabilities were recruited. The participants were invited to join in the group training and they were expected to be able to follow simple instructions in the group activities. Participants who had contraindications to exercise that might influence the response to exercise or with the inability to communicate were excluded. All participants and/or their caregivers were screened by the Physical Activity Readiness Questionnaire (PAR-Q) for their health conditions to ensure they were suitable to participate in exercise training and to determine a starting level for exercise training. The PAR-Q questionnaire contained questions about cardiovascular (heart) condition, chest pain, hypertension, chronic disease, or serious musculoskeletal disorder that would affect the ability to participate in the exercise training. Those with any of these conditions identified were excluded from the study. The residents of the invited centers who passed the PAR-Q screening and had signed the consent form were included in the study. For those who could not sign, consent was obtained from their family members via the social worker of the center.

The participants were divided into the exercise group and control group by cluster sampling. A total of 160 participants from 16 subcenters among five non-governmental organizations completed the video-based exercise program. In addition, 20 participants with intellectual disabilities were recruited as control. The participants of the control group continued with their daily living activities while receiving usual care from their centers with no exercises classes.

### 2.3 Intervention

A video-based exercise program with a series of short duration and moderate intensity exercise sessions with progression over 8 weeks was designed for people with ID to improve their body composition and physical performance. The video comprised rhythmic movements with light-tempo music and animation. The classes were held in the daycare center or hostels, providing a safe and enclosed exercise environment. Participants in groups of 10–15 with 2–3 caregivers/staff participated in each exercise class. Each session lasted for 1 h and comprised the following: (i) 10 min warm up and stretching; (ii) 15 min cardio fitness training exercises; (iii) 10 min muscle strength training; (iv) 10 min balance and coordination training; (v) 10 min fun activities; and (vi) 5 min cool down and stretching. Each participant received training sessions twice a week for 8 weeks. Five video clips were produced with two video clips for the low-level group (LLG) and two videos for the high-level groups (HLG). One video clip was specially made for those who may be more severely disabled with visual or hearing impairments and be wheelchair-bound [labeled as the special-level group (SLG)] Exercise variety included individual exercises, exercise with partners, chair exercise, towel exercise, Ball Fun, and Steps with beats. Registered physiotherapists and physiotherapy students were present at training sessions to supervise the participants and caregivers.

### 2.4 Outcome measures

Physical performance was measured as a primary outcome. Body composition including waist circumference and body mass index (BMI) were measured as secondary outcomes. Assessments were conducted at baseline and at the end of the exercise training. Physical performance was measured by a number of standardized tests that were commonly used in previous research on people with ID (1, 11–14) and are described as follows:

#### 2.4.1 Thirty-second chair stand

The 30-s chair stand repetitions were used to assess participants’ lower limb strength and endurance (13). This test measures the maximum number of times a participant can rise to a full standing position from the seated position in a 30-s period. A standardized unarmed chair was used. The participants were instructed to sit with back straight and feet approximately shoulder-width apart and placed on the floor. Their arms were crossed at the wrists and held against the chest for as long as possible. The maximum number of repetitions achieved was recorded.

#### 2.4.2 Five-time chair stand

The five-time chair stand duration test involves standing up fully from a sitting position and sitting down five times as quickly as possible without pushing off (1). The amount of time a participant took to stand up five times in a row from a seated position on a standard chair with arms folded across the chest was recorded.

#### 2.4.3 Four-meters comfortable walk time

Walking speed was assessed by the 4-m comfortable walk time (1). A 4 m distance was measured and the starting and ending points were marked on the floor. Participants were instructed to walk at their usual pace from a standing position that was normal and comfortable for them until they crossed the finish line. Time was recorded from the first foot movement to the stop timing when the participant's foot made contact with the floor at the end of the walking course.

#### 2.4.4 Six-min walk test

The 6-min walk test was used to assess mobility and submaximal exercise performance. The participants were instructed to walk to and fro on a 10-me walkway on level ground as far as possible. The distance covered by each participant was measured and recorded (12).

#### 2.4.5 Standing static balance level

The standing static balance level was assessed in four increasingly challenging positions (1): (1) feet together (side-by-side stand), (2) instep of foot advanced to toe of other foot (semi-tandem stand), (3) foot in front of other foot (tandem stand), and (4) single-leg stand. Stage success was graded when a participant was able to maintain the position for 10 s, with less than 10 s indicating stage failure. Improvement was demonstrated by completion of more test stages (0–4) at post-intervention testing.

#### 2.4.6 Short physical performance battery score

The short physical performance battery (SPPB) score is a composite score calculated based on the five-time chair stand duration test (for lower extremity strength), 4-m comfortable walk time (for gait speed), and standing static balance level (for standing balance) (1, 14). The performance in each of the three tests was assigned a categorical score ranging from 0 (unable to complete the test) to 4 (highest level of performance) using standardized scoring. The total score ranging between 0 and 12 was calculated.

### 2.5 Body composition

The participants in the exercise group were evaluated in terms of their body mass index (BMI) and waist girth before and after the exercise training. These were also compared with those in the control group at the same time intervals.

### 2.6 Data analysis

Data analyses were conducted using SPSS Statistics 29.0 (IBM, Armonk, NY, USA). Baseline characteristics of the two groups were compared using independent t-test for continuous variables and Chi-square tests for the categorical variables. Differences between groups before and after exercise were evaluated using repeated measures ANOVA. Analyses of covariance (ANCOVA) with baseline as the covariate were used to examine the difference in means between the exercise group and control group on SPPB and the 6-min walk test. Data were analyzed on an “intention-to-treat” basis, with patients being analyzed in the group to which they were assigned. All statistical tests were performed at the level of significance of *P *< 0.05.

## 3 Results

### 3.1 Participants’ characteristics

In this study, a total of 180 participants with intellectual disabilities (160 in exercise group and 20 in control group) were recruited from 16 subcenters from five non-governmental organizations. The demographic characteristics of the participants are provided in Table 1. The participants in this study can be generally of middle age with the mean age being in the mid-40s for the two groups. The mean age in the control group was somewhat higher (49.20 ± 10.91), but their body size measures were largely similar to the exercise group. In terms of their baseline measures in the physical performance tests, there were no significant differences between groups. These results suggest that the unbalanced group sizes did not significantly affect the comparisons of their outcome measures.

**Table 1:** Demographic and clinical characteristics of study participants.

	Exercise group (*n* = 160)	Control group (*n* = 20)	*P*-value
Age (years)^b^	44.59 ± 11.79	49.20 ± 10.91	0.098
Gender (*n*)	Male 95	Male 18	0.006^a^*
	Female 66	Female 2	
Level of intellectual disabilities (*n*)	Mild 41	Mild 6	0.909^a^
	Moderate 108	Moderate 13	
	Severe 10	Severe 1	
Tested positive in COVID-19 (*n*)	123	20	0.015^a^*
Weight (kg)^b^	58.47 ± 9.65	60.33 ± 12.85	0.436
Height (m)^b^	1.59 ± 0.11	1.61 ± 0.09	0.319
Waist girth (cm)^b^	84.55 ± 9.05	81.99 ± 12.18	0.299
Body mass index (kg/m^2^)^b^	23.27 ± 3.44	23.40 ± 5.00	0.930

^a^ Chi square.

^b^ Data expressed as mean ± SD.

* *P *< 0.05.

### 3.2 Physical test performance

The outcomes of the physical tests are presented in Table 2 to compare the baseline and post-intervention measures within group and between groups.

**Table 2:** Physical performance before and after exercises among participants with intellectual disabilities.

		Exercise group	Control group	Between-group *P*-value
30-s chair stand (reps)	Baseline	9.63 ± 3.19	8.90 ± 2.47	0.333
	Post	10.33 ± 3.23	9.65 ± 2.86	0.388
	Within-group *P*-value	0.001*	0.083	
	Overall within-group effects *P *= 0.014; Overall between-groups effect *P *= 0.310; Group-time interaction *P *= 0.937.*			
Five-times chair stand (s)	Baseline	15.47 ± 8.19	16.13 ± 8.19	0.882
	Post-intervention	14.20 ± 6.93	16.23 ± 12.25	0.463
	Within-group *P*-value	0.013*	0.939	
	Overall within-group effect *P *= 0.415; Overall between-group effect *P *= 0.443; Group-time interaction *P *= 0.341.			
4-m comfortable gait speed (s)	Baseline	4.87 ± 2.47	4.88 ± 1.39	0.910
	Post-intervention	4.53 ± 2.05	4.36 ± 1.28	0.731
	Within-group *P*-value	0.008*	0.006*	
	Overall within-group effect *P *= 0.015; Overall between-group effect *P *= 0.870; Group-time interaction *P *= 0.615.*			
6-min walk test (m)	Baseline	312.35 ± 0.00^a^	312.35 ± 0.00^a^	1.000
	Post-intervention	339.84 ± 5.04^a^	324.45 ± 13.83^a^	0.299
	Within-group *P*-value	<0.001^b^*	0.657^b^	
	Overall within-group effect *P *= 0.001; Overall between-group effect *P *= 0.299; Group-time interaction *P *= 0.299. Covariates are evaluated as 6-min walk test (m) = 312.35.*			
SPPB score	Baseline	8.83 ± 0.00^a^	8.83 ± 0.00^a^	1.000
	Post-intervention	9.42 ± 0.13^a^	9.98 ± 0.36^a^	0.286
	Within-group *P*-value	<0.001^b^*	0.003^b^*	
	Overall within-group effect *P *< 0.001; Overall between-group effect *P *= 0.286; Group-time interaction *P *= 0.286. Covariates are evaluated as SPPB = 8.83.*			

^a^ Data expressed as adjusted mean ± SEM.

^b^ Adjusted for multiple comparisons by Bonferroni correction.

* *P *< 0.05.

#### 3.2.1 Thirty-second chair stand

On average, from baseline to after exercise training, there was significant improvement in the 30-s chair stand test (from 9.63 ± 3.19 to 10.33 ± 3.23 repetitions, *P *= 0.001) in the exercise group. The control group also showed slight decrease, but the change was not statistically significant (*P *= 0.083). However, no significant difference was found between groups at post-intervention assessment (*P *= 0.388).

#### 3.2.2 Five-time chair stand

The five-time chair stand test showed a decrease from 15.47 ± 8.19 to 14.20 ± 6.93 s at post-exercise in the exercise group, and this change was statistically significant (*P *= 0.013). No statistical difference was found within the control group (*P *= 0.939) or between two groups at post-intervention assessment (*P *= 0.463).

#### 3.2.3 Four-meters comfortable walk time

Significant improvement in the 4-m comfortable gait speed (from 4.87 ± 2.48 to 4.53 ± 2.05 s, *P *= 0.008, vs. 4.88 ± 1.39 to 4.36 ± 1.28 s, *P *= 0.006) were seen in both the exercise group and the control group, respectively. Yet, there were no statistical differences between the groups (*P *= 0.731).

#### 3.2.4 Six-minute walk test

The 6-min walk test improved from 312.35 ± 0.00 to 339.84 ± 5.04 m in the exercise group and this is a significant change (*P *< 0.001). No statistical difference was found within the control group (*P *= 0.657) or between the two groups at post-intervention assessment (*P *= 0.299).

#### 3.2.5 Static standing balance level

Approximately 26% of the participants showed significant improvement in the 10-s static balance test at post-intervention while 52% remained the same (Chi-square *P *< 0.001) (Table 3). However, the participants in the control group did not show any changes in balance performance after the study period (Chi-square *P *= 0.926).

**Table 3:** Frequency table on 10-s static balance test before and after the exercise program among participants with intellectual disabilities.

			10-s Static balance test (level)—post				Total
			Side-by-side stand	Semi-tandem stand	Tandem stand	Single-leg stand	
Exercise group	10-s static balance test (level)—Baseline	Side-by-side stand	11	9	11	1	32
		Semi-tandem stand	2	12	14	5	33
		Tandem stand	0	7	26	14	47
		Single-leg stand	0	0	4	24	28
	Total		13	28	55	44	140
Control group	10-s static balance test (level)—Baseline	Side-by-side stand	0	0	0	0	0
		Semi-tandem stand	0	0	0	0	0
		Tandem stand	0	0	11	4	15
		Single-leg stand	0	0	3	1	4
	Total		0	0	14	5	19

#### 3.2.6 Short physical performance battery score

Significant improvement in the SPPB score (from 8.83 ± 0.00 to 9.42 ± 0.13, *P *< 0.001, vs. 8.83 ± 0.00 to 9.98 ± 0.36, *P *= 0.003) was seen in both the exercise group and control group, respectively. The between-group differences were not statistically significant at post-intervention assessment (*P *= 0.286).

### 3.3 Body composition

There was a significant decrease in waist girth in the exercise group after the exercise program (from 84.55 ± 9.05 to 83.72 ± 8.59 cm, *P *= 0.015), whereas there was no statistical change in waist girth in the control group (group × time interaction *P *= 0.002) (Table 4). Body mass index remained stable for both the exercise group and control group, before and after the exercise program.

**Table 4:** Body composition before and after the exercises among participants with intellectual disabilities.

		Exercise group (*n* = 160)	Control group (*n* = 20)	Between-group *P*-value
Waist girth (cm)	Baseline	84.55 ± 9.05	81.99 ± 12.18	0.299
	Post-intervention	83.72 ± 8.59	84.25 ± 12.18	0.851
	Within-group *P*-value	*0.015	0.066	
	Overall within-group effect *P *= 0.152; Overall between-group effect *P *= 0.637; Group-time interaction *P *= 0.002.*			
Body mass index (kg/m^2^)	Baseline	23.27 ± 3.44	23.40 ± 5.00	0.930
	Post-intervention	23.38 ± 9.36	23.22 ± 4.42	0.880
	Within-group *P*-value	0.469	0.792	
	Overall within-group effect *P *= 0.872; Overall between-group effect *P *= 0.985; Group-time interaction *P *= 0.539.			

* *P *< 0.05.

## 4 Discussion

It is well known that people with ID have impaired fitness levels due to their sedentary lifestyle and lack of exercise (1, 15). The purpose of this clinical trial is to design a special exercise program that can cater to the different physical abilities of people with ID, and also integrate the elements of music and animation to enhance the compliance of the participants. Training the frontline staff in the residential centers and using the video clips to standardize the exercise training program also helps to ensure that the program is run in a consistent manner across the different centers. Particularly, the exercises were adapted to the different mobility levels of the participants, so it was easier for the participants in the same group to perform the movements required. The results of the present project have demonstrated the effectiveness of such a video-based exercise program that has significantly improved participants’ physical and balance performance.

The significant improvements in the 30-s chair stand and five-time chair stand imply that the participants have gained more muscle strength in their lower limbs, so they can perform better in these tasks. This is also consistent with the faster walking speed in the 4-m gait speed test and the distance walked in the 6-min walking test. The participants have gained more endurance in their walking ability. In a similar trend, their balance performance has also improved as they could perform the various components in the balance testing more successfully. The reduction in waist girth was a good indicator of the exercise effects on improving body composition. Although there was no significant improvement in the body mass index, this may show a more apparent change if the exercise program was continued for longer periods.

The positive results in the present project are generally in agreement with the results reported in past research studies demonstrating the benefits of exercise training on the physical performance or “skill-related fitness” of people with intellectual disability, but there may not be demonstrable benefits in terms of cardiovascular fitness outcomes or body composition measures (9).

Several past studies have reported the benefits of conventional exercise training in terms of aerobic training in improving the cardiovascular fitness and brachial systolic blood pressure in people with intellectual or developmental disabilities (16–18). However, the outcomes were varied and not conclusive. Jo et al. (19) conducted the exercise training for 12 weeks on adults with ID, with only 10 subjects in the exercise group and 10 subjects in the control group. The program included aerobic exercises with background music and conventional strength training with Theraband for 90 min each session, twice a week. Their results did not show significant improvement in muscle mass, strength, and cardiovascular fitness outcomes but significant improvement in muscle endurance (sit-up) (19). A recent study by Melo et al. (16) compared the effects of gym-based vs. home-based multi-component aerobic training regimes on cardiorespiratory fitness on 17 adult participants with ID. Home-based exercise training was most relevant for the COVID pandemic and this was shown to be useful on maintaining central arterial stiffness and central blood pressure, but the overall benefits on cardiovascular fitness were to a lesser extent compared with gym-based training (16). Our current study with the video-based exercise program can be applied both in-person or online, which offers a flexibility to the service units and participants. Our findings are also consistent with these past studies, demonstrating more apparent benefits in skill-related fitness rather than cardiovascular fitness.

Besides aerobic and resistance training, balance training has also been an important component in exercise training for people with ID. In the systematic review by Maiano et al. (8) that evaluated the effects of exercise on balance performance in young people with ID, it was reported that static and dynamic balance performance was significantly better in the exercise intervention group as compared with the control group. The research studies were included from 1991 to 2017 and the majority of the studies involve balance and strengthening exercises in the traditional approach. There were also large variations in the exercise types including creative dance, Tai Chi, Swiss ball, and rope skipping (8). Our present results also showed positive benefits on balance performance in the participants after 8 weeks of a multi-component exercise program.

Indeed, the problem of low physical activity in adults with intellectual disability is a well-recognized problem internationally (15). Particularly, in the current worldwide trend of improved care and standard of living, this group of people is facing the problem of aging. Previous studies reported that older adults with ID had even lower levels of physical activity compared with the normal population of similar age (20, 21). In our present study with the mean age of 40+ years in the exercise group, it was also found that they had lower levels of fitness, which may also be partly due to their experience of COVID-19 in the few months before the exercise program. It is hoped that participating in the exercise program would motivate them to continue with increased physical activity levels in the future.

In the recent research, there has been increasing work to try different types of exercise interventions including gamification and virtual reality technology to attract the people with ID to be more physically active (22). Future studies of randomized controlled experimental design and follow-up assessment are needed to examine the long-term effect and compliance of video-based exercise programs. In conclusion, participation in the exercise program is a key strategy for improving physical and balance performance and could potentially improve the quality of life for people with ID.

### 4.1 Study limitations

The present exercise training program was run for 8 weeks’ period, which has been fixed arbitrarily as there were large variations in previous research studies with training durations ranging from 6 to 28 weeks. The 8-week intervention program has produced significant improvement in some physical performance measures in the exercise group. However, there were no significant between-group differences in the outcome measures, which may be due to the small and unbalanced sample sizes. To produce more effective and long-lasting improvement in cardiovascular fitness and body composition, it would be good to have these training programs adopted as regular activities in the various centers.

Our present study did not have any long-term follow-up after the exercise training was completed and this would be another limitation of the study. It would be useful to re-assess the participants in 3–6 months’ time after the exercise training was completed, to see whether they would continue to exercise, and whether they continued to improve in their fitness and physical performance.

## 5 Conclusion

A specifically designed exercise training program of 60 min, incorporating music and animation captured in short video clips of 15 min have produced positive outcomes in terms of skill-related fitness and balance performance within the exercise group among adults with intellectual disability. A larger-scale study is required in the future to confirm the effectiveness of such a training program with balanced group sizes between the intervention and control groups.

## Data Availability

The raw data supporting the conclusions of this article will be made available by the authors, without undue reservation.
